# Hepatitis C Virus Infection Increases Fatigue in Health Care Workers

**DOI:** 10.3390/diseases8040037

**Published:** 2020-10-15

**Authors:** Vito Emanuele Catania, Giulia Malaguarnera, Giorgia Fiorenza, Eleonora Margherita Chisari, Anna Rita Lipari, Valentino Gallina, Manuela Pennisi, Giuseppe Lanza, Michele Malaguarnera

**Affiliations:** 1 Department of Medical, Surgical Sciences and Advanced Technologies “G.F. Ingrassia”, University of Catania, 95124 Catania, Italy; vito.catania@policlinico.unict.it; 2 “The Great Senescence” Research Centre, University of Catania, 95100 Catania, Italy; giulia.malaguarnera@live.it (G.M.); giorgiaf1d@gmail.com (G.F.); 3 Department of Education, University of Catania, 95100 Catania, Italy; g.chisari@tin.it; 4 SPRESAL ASP ENNA, 94100 Enna, Italy; arlipari@tiscali.it (A.R.L.); valentino.gallina@unikore.it (V.G.); 5 Faculty of Engineering and Architecture-Risk analysis and work safety organization-Kore University of Enna, 94100 Enna, Italy; 6 Department of Biomedical and Biotechnological Science, University of Catania, 95123 Catania, Italy; manuela.pennisi@unict.it; 7 Department of Surgery and Medical-Surgical Specialties, University of Catania, 95123 Catania, Italy; glanza@oasi.en.it

**Keywords:** HCV, fatigue, healthcare workers

## Abstract

Fatigue is a common state associated with a weakening or depletion of one’s physical and mental resources, that leads to the inability to continue the individual functioning at a normal level of activity. Frequently, fatigue represents a response to infections, inflammation and autoimmune diseases. The scope of this study was to evaluate the fatigue in healthcare workers with and without hepatitis C virus (HCV) infection. Mental, physical and severity fatigue were evaluated through Krupp, Wessely and Powell fatigue scale. Anti-HCV antibodies, HCV RNA and HCV genotypes were also measured. Physical, mental and severity fatigue were higher in healthcare workers with HCV infection than the healthcare workers without infection (*p* < 0.01). Our data showed a direct link between fatigue and HCV infection in healthcare workers. Further studies are needed to evaluate HCV antiviral treatments on fatigue severity and on quality of life in healthcare workers

## 1. Introduction

The World Health Organization (WHO) has reported that 71 million persons were living with chronic viral infection worldwide in 2015 [1]. Among these infections, viral hepatitis causes more than a million deaths each year. The global prevalence of hepatitis C virus (HCV) infection is estimated at 2.5%, ranging from 2.9% in Africa to 1.3% in America [2]. The number of individuals chronically infected with HCV is estimated to be approximately 160 million, but most of them are unaware of their infection [3]. HCV infection has increasingly become a public health concern in developed and developing countries. Chronic HCV infection is the primary cause of hepatocellular carcinoma, which is one of the most common cancers and the third cause of cancer-related mortality worldwide [4].

Nowadays it is estimated that about 2% to 4% of the total number of hepatitis C cases are healthcare workers who have suffered a needlestick injury and develop hepatitis as a result [5,6]. The estimated prevalence in UK primary care workers ranges from 0.6 to 2.6% while an independent working party, reporting to the IK Chief Medical Officer in 2002 suggested a UK population prevalence of 0.2–0.4% [7]. In 2007 Safe Work Australia reported that paramedics have the sixth highest rate of occupational injuries [8,9,10]. The principal routes of HCV infection were blood transfusion, unsafe injection procedures and intravenous drug use, but the most common causes reported are due to difficulties in operating the safety device and continued improper disposal of needles [11,12]. Currently screening of blood products for HCV has virtually eradicated transfusion-associated hepatitis C. Occupational exposure affects healthcare workers not only physically but also psychologically. In fact, healthcare workers are exposed not only to risk factors, including physical, chemical, accidentalities and related hazards also both psychosocial and organisational factors [13,14].

Fatigue is a complex and non-specific phenomenon characterized by self-reported tiredness or lack of energy, with a relevant impact on daily activities, social life and work performance [15]. It is also a common symptom in primary care workers [16]. Fatigue is difficult to characterize and define because it encompasses a complex interaction between biological, psychosocial and behavioural processes.

The prevalence of fatigue and its exacerbation known also as chronic fatigue syndrome is difficult to quantify due to the different criteria used [17,18]. It has been estimated to be between 0.24% [19] and 2.55% [20] among adults in the USA. Chronic fatigue syndrome prevalence is estimated at 0.3 to 1% among the public while its prevalence is almost twice as high among health care workers [21]. These workers gradually feel that they can no longer work effectively with their patients and feel difficulties in providing caring duties.

The most common types of fatigue are central (mental) and peripheral (physical). Central fatigue is characterized by a lack of self-motivation and can manifest both in physical and mental activities [22]. Peripheral fatigue is evidenced by neuromuscular dysfunction and muscle weakness [23]. There are many factor that influence fatigue such as age, culture, comorbidities, pain, mood, sleep, affect, anxiety and depression [24].

The pervasive and fluctuating nature of physical and emotional symptoms reduce work performance [25,26]. The quality of care giving services is decreased due to the higher probability of errors caused by occupational fatigue [27]. There is a complex interaction of predisposing precipitating and perpetuating factors in the aetiology of fatigue and support the hypothesis that pre-existing health issues, lack of fitness, distress and stress predisposes to be vulnerable to post infections fatigue. The risk occupational exposure to blood borne pathogens (including hepatitis B, hepatitis C and HIV) via sharps injuries, among health care workers is a challenging issue [28]. The aim of this study was to evaluate relationship between HCV and fatigue in health care workers.

## 2. Patients and Methods

**Figure 1:**
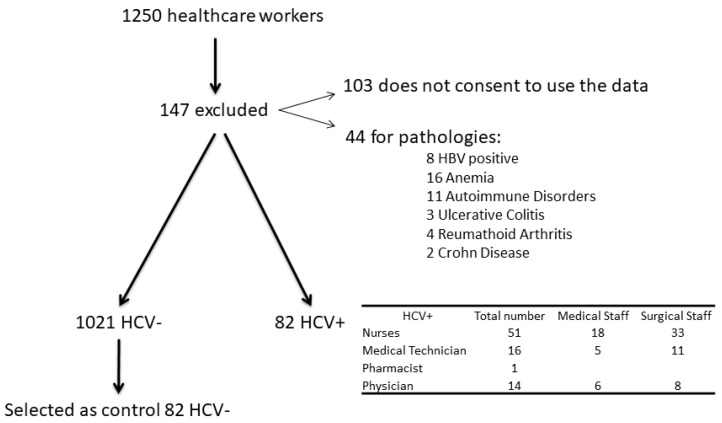
Flowchart.

The study was designed as a cross-sectional controlled study trial. It was performed at the research Centre of Senescence, University of Catania, and PRESAL ASP Enna, between February 2010 and July 2016. One thousand two hundred and fifty (1250) healthcare workers were enrolled of which 147 were excluded from the study. The subjects who did not authorize the use of their data were 103 and another 44 were excluded because they suffered from an autoimmune disease, anaemia or were HBV positive. The final number of healthcare worker were 1103, among them 82 healthcare workers were HCV+ (51 nurses, 16 medical technicians, one pharmacist, 14 physicians; Figure 1–flow chart). The control group consisted of health care workers without viral infection. The initial participants of these group were 1021, then 82 subjects were selected and matched for age and gender, occupational levels and care units in order to be comparable with the HCV group. The study included 164 healthcare workers: 82 with HCV infection and 82 without HCV. HCV infection was confirmed by the presence of HCV RNA detected by polymerase chain reaction (PCR) using Cobas Amplicar testing.

A total of 164 healthcare workers aged 40 to 55 years were included in this study. The demographics and descriptive characteristic of recruited subjects are given in Table 1. Out of the 82 subjects with HCV infection 46 were females and 36 males, with an average age of 46.8 ± 4.1 years. Among the 82 controls, 46 were females and 36 were males, with an average age of 44.7 ± 5.8 years. 

Subjects were required to fast over a period >8 h, follow a standard institutional protocol and were examined after a wash out period of three days. All the subjects were instructed to try to minimize stress variations (anxiety and depression). 

The managers of the units granted permission to collect data and talked to healthcare workers in their units about this project. Eligible workers who wanted to participate were assigned a copy of the study questionnaire. They were instructed to fill it in within three days and return to a box provided for this purpose. The collected data did not contain any names, description or personal data that could identify any of these participants. The results were treated collectively due to the number of participants. The participants worked in different care units including the emergency department at the time of the study.

Previous unsuccessful therapy attempts with less effective treatment’s protocols (e.g., IFN monotherapy) were not an exclusion criterion. All participants were seronegative for hepatitis B surface (HBs) antigen and for human immunodeficiency virus (HIV; type 1 and 2).

HCV-infected populations must have elevated serum alanine transaminase levels of more than 1.5 times upper normal limit and findings on liver biopsy consistent with chronic infection. Ineligible patients were those who had other liver diseases, as well as those who were affected by cancer, severe jaundice, pulmonary and renal chronic diseases, diabetes mellitus, hemoglobinopathies, pregnancy, prostatic and autoimmune diseases, cardiopathy, hemoglobinopathies, hemochromatosis, major depression or other severe psychiatric pathological conditions. None of the patients made excessive use of alcohol (> 20 g/day) or hepatotoxic drugs.

Clinical evaluations, hematochemical, virologic and instrumental were performed on these patients. The study was approved by ethical committee of “The Great Senescence” research centre.

All subjects underwent a physical examination and medical interview before treatment. Study recruitment was performed in observation and respect of the Helsinki Declaration. All participants gave their informed consent for the study participation and for each invasive procedure they underwent.

### 2.1. Serum Analysis

Venous blood samples were collected from all subjects enrolled in the study. Sera were separated, divided into aliquots and stored at −80 °C (until used). All patients underwent a complete virologic assay for HBV and HCV. Hepatitis B surface antigen (HBsAg), hepatitis B “core” IgG antibody (anti-HBc IgG), and hepatitis B “e” antigen (HBeAg), hepatitis B “e” antibody (HBeAb), hepatitis B virus DNA (HBV-DNA) and Delta virus antibody (anti-Delta) assays were performed. Anti-HCV antibodies were determined by enzyme-linked immunosorbent assay (ELISA) using assay kits (Ortho Diagnostic Systems, Raritan, NJ, USA). Hepatitis C virus RNA (HCV-RNA) levels were detected by polymerase chain reaction (PCR) of HCV-RNA 5” UTR using COBAS AmpliPrep/COBAS TaqMan (Roche Diagnostics Systems, Branchburg, NJ, USA). Serum samples negative for HCV RNA were retested using a more sensitive standardized qualitative PCR assay with a lower limit of detection of about 100 IU/mL to confirm HCV-RNA disappearance. HCV genotypes and subtypes were identified [29]. HCV viral genotypes were determined by restriction analysis of HCV-RNA 5”UTR [30]. Aspartate aminotransferase and alanine aminotransferase (AST and ALT), gamma glutamyl transferase (γGT), total, conjugated and unconjugated bilirubin, serum proteins analysis were performed. All liver function tests, hematochemical measurement and virologic analysis have been executed in the laboratory of our hospital using automated and standardized methods.

### 2.2. Questionnaire Evaluating Fatigue

All demographic and clinical data were collected and recorded by trained operators. Weight and height were measured without shoes. Body mass index (BMI) was calculated by the height squared in meters (Kg/m^2^). The participants also completed a self-reported questionnaire.

Fatigue severity was measured by the fatigue severity scale (FSS). The FSS is a self-assessed nine-question scale, each item ranging from 1 (strongly disagree with the statement) to 7 (maximum agreement). Here, the total score ranged from 9 to 63, and it was directly related to the severity observed on the nine-item fatigue questionnaire [31]. The Wessely’s test and Powell’s test were used to examine mental and physical fatigue, respectively. Their scores consisted of a total list of 14 questions, divided in two scales measuring physical fatigue (eight items scored from 0 (no fatigue) to 2 (highest possible fatigue), total score range 0–16); and mental fatigue (five items, total score range 0–10) [32,33,34]

### 2.3. Statistical Analysis

Data were described as mean ± standard deviation (SD) for normally distributed variables and analyzed by unpaired two tailed *t*-tests. Proportion were valued with Fisher‘s exact test and 95% CI of odds ratio (OR) with the Baptista-Pike method. A *p* value <0.05 was evaluated as statistically significant. Data were analyzed using the GraphPad Prism 8 statistical software package (8.4.2 Macintosh Version; GraphPad Software, San Diego, CA, USA).

## 3. Results

The demographics and descriptive characteristic of the recruited subjects are given in Table 1 and Table 2. The comparison of clinical parameters between health care workers with and without HCV is shown in Table 2.

The healthcare workers with HCV showed higher levels of urea (95% CI 0.58 to 5.22 mg/dL), AST (95% CI −131.1 to −120.9 IU/L), ALT (95% CI −140.7 to −126.7 IU/L), CRP (95% CI −4.90 to −4.26 mg/L) (*p* < 0.0001), total cholesterol (95% CI −9.12 to −0.88 mg/dL), HDL cholesterol (95% CI −4.23 to −0.37 mg/dL), and triglycerides (95% CI −9.22 to −0.98) (*p* < 0.05) and lower levels of glucose (95% CI 0.58 to 5.22 mg/dL) (Table 3).

The comparison of mental, physical and severity fatigue in healthcare worker with and without HCV infection is expressed in Table 4. Physical fatigue was higher of 3.2 score in HCV subjects than in health workers without HCV infection (95% CI −4.21 to −2.19; *p* < 0.0001), mental fatigue was higher 1.8 score than in health workers without HCV infection (95% CI −2.76 to −0.84; *p* = 0.0003). Fatigue severity was higher 13.9 score respect subjects without HCV infection (95% CI −16.38 to −11.42; *p* < 0.0001).

## 4. Discussion

HCV positive healthcare workers showed higher fatigue score severity both mental and physical than subjects without HCV infections. Fatigue is a multi-factorial symptom that may be influenced by a variety of demographic, medical, psychosocial, behavioral and biological factors. It has a negative impact on work, social relationship, mood and daily activities and causes significant impairment in overall quality of life. This cross-sectional study confirms the negative impact of HCV infection on physical and mental fatigue (*p* < 0.01) in healthcare worker. Fatigue at work may be associated with decreased vigilance, motivation work capability and performance.

HCV infection leads to chronic liver diseases, such as cirrhosis and hepatocellular carcinoma (HCC). Hepatitis C infection has a negative impact on patients’ quality of life both physically, mentally, emotionally and socially; the effects of hepatitis C extend far beyond liver-related morbidity [35]. In our study, we observed that the HCV positive healthcare workers showed an increase of fatigue. Fatigue severity does not appear to correlate with the biochemical parameters and the subtypes of hepatitis C viruses. Other potential contributing factors include medical comorbidities, medications, nutritional issues, physical deconditioning, mood disturbance and physical symptoms, among others. Fatigue may be influenced by age, culture, comorbidities, pain, mood, sleep and affects [24]. This suggest that several contextual factors may influence the experience of this symptom [36]. Fatigue is a major related factor in healthcare, directly affecting performance, care giving and decision making, resulting in a breakdown in relations between the healthcare and patients, family and team members. It increases mistakes in the administration of medicines and has an impact on practices involving patient monitoring [37,38]. Several studies suggest that people with HCV may have a lower quality of life due to depression, chronic fatigue, fibromyalgia and anxiety in comparison to the general population. Previous studies indicate an increase of depression score, anxiety and fatigue in non-treated HCV patients [39,40]. The disorders above result in higher rates of risking-taking behaviour among the infected when left untreated, thus representing a danger not only to patients themselves, but also to the healthy population.

Fatigue in liver disease is recognised as prevalent and persistent. The liver is central to the pathogenesis of peripheral and central fatigue, which in our view is dependent upon energy regulation and crosstalk between the gut, liver, muscle and brain. The liver is connected to extra-hepatic tissues in order to signal energy needs (skeletal muscle, brain), storage (adipose tissue) and substrate (gut). The liver communicates with extra-hepatic tissue, and with respect to fatigue it communicates through neuronal and hormonal networks.

Different studies suggest that people with HCV may have a lower quality of life due to depression, chronic fatigue and anxiety in comparison to the general population [41,42] Moreover, there is evidence that displays how HCV may affect the central nervous system by generating inflammatory cytokines and toxic substances in the liver that generate symptoms of fatigue and other behavioural changes via alterations in neural processes [43,44,45].

The toxic substances that can be produced include ones like nitric oxide and stimulating amino acids (aspartates and gentamates) which induce neuronal apoptosis and also neurosteroids that activate inhibitory brain pathways [46,47]. It is plausible that inflammatory cytokines serve as mediators of the both environmental and genetic factors that may trigger the development of depressive disorders.

## 5. Conclusions

The cross-sectional nature of this study prevented the establishment of a causal relationship between variables and represents a potential limitation. Therefore, longitudinal studies should be conducted in the future.

The continuous development of health care delivery will be accompanied by the evaluation of new occupational hazards, so to minimize occupational exposure, there is the necessity to implement effective protective measures [48]. The overall impact of HCV treatment with more efficacious and effective direct-acting antiviral (DAA) therapies may be useful for evaluate the direct and indirect role of HCV infections on fatigue [49].

## Figures and Tables

**Table 1:** Gender and lifestyle factors.

	HCV N = 82		NHCV N = 82		HCV vs. Not HCV		
	N	%	N	%	95% CI Odds Ratio	Signif.	*p*
Gender F/M	46/36	56.10	45/37	54.88	0.5805 to 1.905	NS	>0.9999
Smokers Y/N	50/32	60.98	44/38	53.66	0.7421 to 2.484	NS	0.4300
Alcohol Y/N	5/77	6.10	7/75	8.54	0.2401 to 2.118	NS	0.7657
MS Single Y/N	12/70	14.63	10/72	12.20	0.5250 to 3.103	NS	0.8193
MS Married Y/N	60/22	73.17	56/26	68.29	0.6617 to 2.470	NS	0.6069
MS Divorce Y/N	6/76	7.32	10/72	12.20	0.1954 to 1.522	NS	0.4308
MS Widow Y/N	4/78	4.88	6/76	7.32	0.2009 to 2.567	NS	0.7461

HCV = Subjects with HCV virus; NHCV = Subjects without HCV; MS = Marital Status; OL = Occupational Level; NS = Not Significant.

**Table 2:** Demographics and clinical characteristics. Summary *p* value: ** < 0.005; **** < 0.0001.

	HCV N = 82		Not HCV N = 82		HCV vs. not HCV		
	Mean	SD	Mean	SD	95% CI	Significance	*p*
Age (years)	46.8	4.1	44.7	5.8	−3.649 to −0.5511	**	0.0021
Sistolic Art Press (mmHg)	137.1	9.4	138.2	8.7	−1.693 to 3.893	NS	0.4877
Diastolic Art press (mmHg)	81.4	9.1	80.6	9.2	−3.622 to 2.022	NS	0.9219
HR (bpm)	70.2	9.6	72.8	8.7	−0.2253 to 5.425	NS	0.3775
BMI (Kg/m^2^)	24.2	2.9	24	3.1	−1.126 to 0.7257	NS	0.5497
HCV RNA	3.5	0.9	0	0	−3.696 to −3.304	****	<0.0001
HCV exposure (years)	11.2	3.2	/	/			
Anti HCV Ab	positive		negative				
HCV genotypes							
1a	10	/	/	/			
1b	69	/	/	/			
2a	1	/	/	/			
2b	2	/	/	/			

Summary *p* value: ** <0.005; **** <0.0001.

**Table 3:** Laboratory parameters.

	HCV N = 82		Not HCV N = 82		HCV vs. Not HCV		
	Mean	SD	Mean	SD	95% CI	Signif.	*p*
Urea mg/dL	44.1	3.7	38.1	4.1	0.5829 to 5.217	****	<0.0001
Glucose mg/dL	74.2	8.7	77.1	6.1	0.5829 to 5.217	*	0.0145
Total Cholesterol mg/dL	225	12.8	220	13.9	−9.121 to −0.8794	*	0.0177
HDL Cholesterol mg/dL	44.1	6.2	41.8	6.3	−4.228 to −0.3724	*	0.0197
Triglycerides mg/dL	190.1	12.8	185	13.9	−9.221 to −0.9794	*	0.0156
AST IU/L	164.2	22.9	38.2	3.9	−131.1 to −120.9	****	<0.0001
ALT IU/L	169.8	32.1	36.1	3.4	−140.7 to −126.7	****	<0.0001
CRP mg/L	6.25	1.44	1.67	0.36	−4.904 to −4.256	****	<0.0001

Summary *p* value: * < =0.05; **** <0.0001.

**Table 4:** Fatigue results.

	HCV N = 82		Not HCV N = 82		HCV vs. Not HCV		
	Mean	SD	Mean	SD	95% CI	Signif.	*p*
Physical fatigue	11.4	2.9	8.2	3.6	−4.208 to −2.192	****	<0.0001
Mental Fatigue	9.7	3.4	7.9	2.8	−2.761 to −0.8395	***	0.0003
Fatigue Severity	49.1	6.7	35.2	9.2	−16.38 to −11.42	****	<0.0001

Summary *p* value: *** < 0.001; **** <0.0001.
